# Electroless Deposition for Robust and Uniform Copper Nanoparticles on Electrospun Polyacrylonitrile (PAN) Microfiltration Membranes

**DOI:** 10.3390/membranes14090198

**Published:** 2024-09-20

**Authors:** Temitope Q. Aminu, Hamid Fattahi Juybari, David M. Warsinger, David F. Bahr

**Affiliations:** 1 School of Materials Engineering, Purdue University, West Lafayette, IN 47907, USA; 2 Birck Nanotechnology Center, Purdue University, West Lafayette, IN 47907, USA; 3 School of Mechanical Engineering, Purdue University, West Lafayette, IN 47907, USA

**Keywords:** microfiltration, electroless deposition, electrospinning, nanocopper, polyacrylonitrile fibers

## Abstract

Filtration membranes coated in metals such as copper have dramatically improved biofouling resistance and pathogen destruction. However, existing coating methods on polymer membranes impair membrane performance, lack uniformity, and may detach from their substrate, thus contaminating the permeate. To solve these challenges, we developed the first electroless deposition protocol to immobilize copper nanoparticles on electrospun polyacrylonitrile (PAN) fibers for the design of antimicrobial membranes. The deposition was facilitated by prior silver seeding. Distinct mats with average fiber diameters of 232 ± 36 nm, 727 ± 148 nm and 1017 ± 80 nm were evaluated for filtration performance. Well-dispersed copper nanoparticles were conformal to the fibers, preserving the open-cell architecture of the membranes. The copper particle sizes ranged from 20 to 140 nm. Infrared spectroscopy revealed the PAN fiber mats’ relative chemical stability/resistance to the copper metallization process. In addition, the classical cyclization of the cyano functional group in PAN was observed. For model polystyrene beads with average sizes of 3 μm, Cu NP–PAN fiber mats had high water flux and separation efficiency with negligible loss of Cu NP from the fibers during flow testing. Fiber size increased flux and somewhat decreased separation efficiency, though the efficiency values were still high.

## 1. Introduction

Filtration membranes based on electrospun fibers of synthetic polymers have gained widespread acceptance. These membranes provide tailorable, low-cost, and energy-efficient alternatives to membranes derived from mainstream processes such as phase inversion, template synthesis, and drawing [1,2]. The nonwoven fiber architecture traditionally generated by fiber accumulation from the electrospinning process possesses hallmark structural features that facilitate effective filtration: a porous nature (open cell), constituent fibers with high surface area to volume ratios, and characteristic dimensions (of pores and fibers) that are on the same order or scale as the particulate matter to be filtered [2,3]. Furthermore, the minimal efficiency of electrospun fiber membranes has been shown to increase with decreasing fiber size [2], due to an increasingly convoluted topology of fiber networks coupled with interception effects.

For water purification applications, microfiltration is used for the removal of organic and inorganic particulate matter, with typical membranes pore sizes in the range of 0.1–10 μm [4,5]. In particular, electrospun microfiltration membranes have been touted as high flux, high permeability microfiltration candidates that can be integrated into water purification systems to serve as an upstream pretreatment step for more sophisticated treatment processes [6], or as point-of-use purification to enhance potable water quality [4].

The pollution of drinking water by pathogenic bacterial contamination has been identified as the greatest water-borne threat to human health [7]. Given the omnipresence of these microorganisms, complete elimination may be difficult to achieve unless antimicrobial treatment processes are deployed at points of water dispensation to achieve a high level of sterility. Advantageously, the typical size range of pathogenic bacterial cells (0.1–10 μm) falls within the microfiltration size exclusion regime [3], and through the inherent tunability of the electrospinning process the pore sizes of electrospun microfiltration membranes can be optimized for selectivity or specificity. Additionally, immobilization of active functional species on the fiber surface, e.g., charged groups or ligands, may promote the capture of even smaller particles (outside of the intrinsic size exclusion regime) through surface adsorption processes [8,9].

The effects and utility of metal-based antimicrobial materials have been historically established, but contemporary adoption is not widespread. Antimicrobial metallic materials like copper and silver are cytotoxic to pathogenic microbes at very low concentrations, while being physiologically tolerable or innocuous to humans [10,11]. At nanoparticulate (NP) sites, copper and silver possess strong antibacterial properties. This facilitates microbial destruction or deactivation through the release of toxic ions, ultimately promoting membrane-disruptive processes and encouraging the proliferation of harmful reactive oxygen species in the cellular environment [10].

The functionalization of electrospun fibers with Cu NPs and Ag NPs for filtration membranes has been achieved through a wide range of strategies. A common processing scheme involves mixing the colloidal solutions of either Cu NPs or Ag NPs within the polymer solution prior to electrospinning [12,13]. However, the immobilization/deposition of nanoparticles on the fiber surface is usually preferable for more active surface areas. Such surface coatings typically involve the grafting of pre-formed Ag NPs with functional chemical groups or by photocatalytic reduction in adsorbed silver ions [14,15]. For Cu NPs, deposition can be achieved through sequential immersion in solutions of a copper salt and a reducing agent [14]. Alternatively, Cu NPs of distinct speciation have also been incorporated into electrospun fibers by mixing varying concentrations of copper salts with the polymer solution without the use of reducing agents or post-processing [16]. Electroplating can also create metal coatings on membranes but faces challenges on polymer membranes, as the electric current does not conduct well through the polymers’ chaotic structures. Unfortunately, most of these methods have problems achieving uniform coatings. In contrast, electroless deposition, which uses an autocatalytic reaction instead of an electric current in the reduction in metal ions, can coat uniformly through the filter thickness.

The electroless method offers functionalization of electrospun polymer fibers with a simple but versatile pathway for the direct growth of metallic nanostructures exclusively on the fiber surface, affording control over morphology, size, shape, and density of the resultant structures [17,18]. Electroless deposition is predicated on solution precipitation enabled by spontaneous electrochemical reactions. For nonconductive substrates such as polymer fibers this is typically catalyzed using seed crystals of noble metals like palladium or silver [19,20]. Furthermore, our previous work has demonstrated that electroless polymeric fiber functionalization with metallic nanostructures promotes good interfacial adhesion between the polymer and metal phases, ensuring the integrity of the material design [17]. With respect to Cu NP deposition, the use of an electroless process for coating polymeric filtration membranes has been limited. The electroless deposition of Cu-NP on a PET ion-track membrane was investigated, wherein a select number of reducing agents were evaluated [21]. Cu NPs were also electrolessly deposited on closed-cell, reverse osmosis membranes [22,23]. However, the deposition kinetics was slow (~2 h) and a substantial drop in flux was observed. In addition, the nanoparticles agglomerated in clusters on the membrane structure, limiting dispersion and preventing a fully integrated material design. Electrospun nanofiber mats with conformal copper nanoclusters for oil–-water emulsion separation were fabricated by a silver seed-assisted deposition of copper [24]; but the silver seeds were induced by microwave irradiation of silver precursors which may not afford tight controls or tuning of the seeding process and subsequent electroless deposition.

In this work, we have used electroless deposition to immobilize copper nanoparticles on electrospun polyacrylonitrile fiber mats catalyzed by silver, evaluating the potential for use of the resultant nanocomposite architecture as antimicrobial filtration membranes. Silver seeds promote the dehydrogenation of formaldehyde in electroless copper baths, creating active reduction species that ultimately induce deposition of copper [25]. To the best of our knowledge, no studies have been implemented on the fabrication of electrospun antimicrobial filtration membranes with electrolessly deposited copper nanoparticles. At best, previous studies have generated membranes by electrospinning the mixture of preformed (ex situ) Cu-NPs and the polymer solution [26,27], leading to a substantial amount of functional nanoparticles embedded in the polymer fiber [25]. Polyacrylonitrile is a thermoplastic polymer with good thermal stability, chemical resistance, and robust mechanical properties, as well as being amenable to modifications prior to and after the electrospinning process [28]. Additionally, it has been used extensively in the fabrication of microfiltration membranes for water purification [8,29,30]. While other methods of incorporating metallic NPs are possible, such as spray/spin-assisted deposition [31], solution deposition of NP suspensions [32], and reviews covering a broad range of techniques [33], we focused on electroless deposition for its speed and scalability. Therefore, our goal with this study was to determine if it is possible to deposit Cu NPs with a discrete distribution on three electrospun mats with distinct fiber sizes ranging from submicron to micron dimension, where pore sizes are in the microfiltration regime, and characterize the stability of the membranes and performance in filtration. The membrane displayed high water flux and high separation efficiency for model particles of size 3 μm.

## 2. Experimental Methods

Details of fiber membrane fabrication, the electroless deposition process, material characterization, and flux and filtration measurements are provided below.

### 2.1. Electrospinning: Fibrous Membrane Production

PAN powders (Sigma-Aldrich, M_w_ = 150,000 g/mol, ρ = 1.18 g/cm^3^) were dissolved in dimethylformamide (DMF) to make solutions with 9 wt%, 11 wt%, and 13 wt% PAN concentration. Subsequently, 1 wt% acetone was added to the resulting solutions. Acetone has been shown to mitigate or eliminate bead formation during electrospinning [34]. The solution vessel was wrapped in aluminum foil and stirred continuously for 24 h at room conditions. The electrospinning solution was loaded into a 3 mL syringe with a 19 ga (ID = 0.8126 mm) needle. A 13 × 13 cm^2^ piece of aluminum foil, situated at a distance of 15 cm from the syringe tip, was the designated collector plate. A voltage generator (Model SL300, Spellman High Voltage Electronics, Hauppauge, NY, USA) supplied a constant DC voltage of 15 kV between the collector plate and needle tip, creating an electric field strength of 100 kV/m. A flow rate of 0.34 mL/h was supplied by a syringe pump (Advance infusion pump series 1200). The total fiber deposition time was 8 h. Coupons for pore size measurement, flux, and filtration efficiency measurement were cut from the aluminum foil and subsequently peeled off. The average mat thickness was 30 μm. The areal densities of the mats were in the order of 10 g/m^2^. This process flow is shown in Supplemental Figure S1.

### 2.2. Membrane Preparation for Electroless Deposition

For each fiber diameter, 5 cm by 5 cm mats were cut for electroless deposition. All sides of the samples were attached to rectangular strips with adhesive carbon tape to ensure the mats were flat during the chemical treatment in the aqueous baths. All samples were pre-cleaned in a 1.63 M solution of soda ash (Na_2_CO_3_) for 3 min at room temperature. Subsequently, the mats were treated in a 1 M solution of sodium hydroxide (NaOH) at a temperature between 45 and 50 °C for 15 min. For silver seeding, an initial solution containing 200 µL of ammonia solution (NH_4_OH) and 10 mL of 0.01 M AgNO_3_ was prepared under constant stirring (≈200 rpm). In total, 5 mL of 10 wt. % glucose (C_6_H_12_O_6_) was added to this solution and stirred for 1 min. To prevent the premature reduction in silver ions by photocatalysis, the reaction vessel was wrapped with aluminum foil. Samples were immersed in the silver baths for 1 min and subsequently rinsed with a copious amount of deionized (DI) water. Each fiber mat seeding was carried out in a fresh bath. The seeding procedure was carried out at room temperature under quiescent conditions. All chemicals were reagent grade.

### 2.3. Electroless Copper Deposition

A total of 0.4 mmol of copper sulfate pentahydrate (CuSO_4_.5H_2_O) crystals was mixed with 1.6 mmol of disodium ethylene diamine tetraacetate (Na_2_H_2_EDTA.2H_2_O) in 20 mL of deionized water. Subsequently, 280 μL of formaldehyde (HCHO) was added to the solution. Droplets of a 1.15 M solution of sodium hydroxide were added to the solution until the pH of the metastable bath was 12.3–12.4, monitored using a pH meter (Fisherbrand AB15, Thermo Fisher Scientific, Waltham, MA, USA). The mats were immersed in the solution for 15 min, whereupon they were thoroughly rinsed in deionized water and air dried.

Silver seeding reaction [25]:

(1) $${{Ag}^{+}}_{\left(aq\right)}+{{2NH}_{3}}_{\left(aq\right)}⇌{Ag{\left(NH_{3}\right)}_{2}^{+}}_{\left(aq\right)}$$

(2) $${2\left[Ag{\left({NH}_{3}\right)}_{2}\right]}^{+}+R-CHO(glucose)+H_{2}O⇌{{2Ag}^{0}}_{\left(s\right)}\left(seeds\right)+{2NH}_{3}+R-COOH+2H^{+}$$

Electroless copper precipitation reaction [25]:

$${Cu}^{2+}+2e⟶Cu$$

$$HCHO+{2OH}^{-}⟶{HCOO}^{-}+H_{2}O+{\frac{1}{2}H}_{2}O+e$$

$${Cu}^{2+}+2HCHO+{4OH}^{-}\;⟶\;Cu+2{HCOO}^{-}+2H_{2}O+H_{2}$$

### 2.4. Characterization

The fiber mat microstructures were characterized with a FEI NovaNano200 scanning electron microscope (SEM) (ThermoFisher Waltham, MA USA). Samples were sputter-coated with a thin (≈2 nm) layer of platinum in a Cressington (model 208HR, Cressington Scientific Instruments, Watford WD19 4BX, UK) coater at a plasma current of 40 mA for 1 min to minimize charging effects. Electron dispersive X-ray analysis (EDX) was performed using an Oxford INCA energy 250 system (Oxford Instruments, High Wycombe, UK). Phase identification was evaluated using X-ray diffraction in a Bruker D8 Focus system (Bruker AXS, Karlsruhe, Germany) operating at 40 kV and 40 mA. The scan speed was 5°/min, spanning a range of 2ϴ angles from 10° to 80°. Polymer chemistry was investigated using Fourier transform infrared spectroscopy (FTIR) (ARCoptix, Neuchatel, Switzerland), performed using Perkin Elmer IR 100 spectrometer (Perkin Elmer, Shelton, CT, USA) operated in transmittance mode and spanning wavenumbers from 4000 to 500 cm^−1^ at room conditions.

### 2.5. Pore Size Measurement

The average pore size, pore size distribution, and bubble point were measured using a capillary flow porometer (Porous Materials Inc. (PMI). Model CFP 1500AL, Ithaca, NY, USA). Each sample was cut to a 2.5 cm diameter and wetted with Galwick (surface tension 15.9 dynes/cm, provided from PMI). Its working principle is based on the gas–liquid displacement and gas permeation. Each sample ran three times, and an average was reported here.

### 2.6. Water Flux and Separation Efficiency Measurement

**Figure 1:**
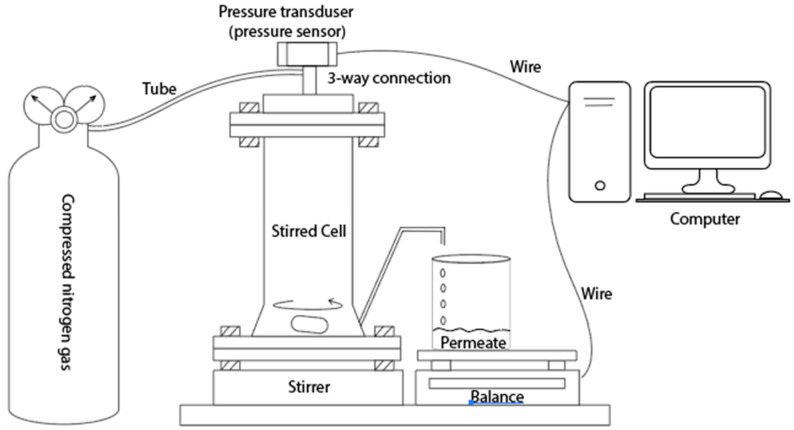
Dead-end membrane filtration setup.

A stainless steel dead-end stirred cell (HP4750 Stirred Cell, Sterlitech Corp., Kent, WA, USA) setup, as seen in Figure 1, with a 2.3 cm^2^ active membrane surface area was used for measuring the permeate flux and separation factor (SF) of the electrospun membranes. The dead-end cell was filled with 100 mL DI water and compressed (5 psi) with nitrogen before collecting 50 mL permeate for the DI water flux measurement. The permeate weight was measured using a computer-controlled balance. For the filtering test, polystyrene beads (Sigma-Aldrich, St. Louis, MI, USA) with a mean particle diameter of 3 μm were diluted to 100 ppm in distilled water. The filtering test used the same approach as the DI water flux measurement. To determine the effect of the filtration test on the membrane flux, the used membrane was rinsed with DI water three times, and then the DI water flux was measured. Each sample was examined three times, and the average flow measured is reported here. For the calculation of separation factor and particle concentration in the permeate, a UV–Vis spectrophotometer (UV-2700, Shimadzu Corp., Kyoto, Japan) was used. A calibration curve was generated by measuring the solution absorbance at pre-determined concentrations. The separation factor (SF) is calculated from the following:

$$SF=\left(1-\frac{C_{permeate}}{C_{feed}}\right)\times 100\%$$

$C_{permeate}$ and $C_{feed}$ are the polystyrene bead concentrations in the permeate and feed, respectively.

## 3. Results and Discussions

### 3.1. Microstructure

**Figure 2:**
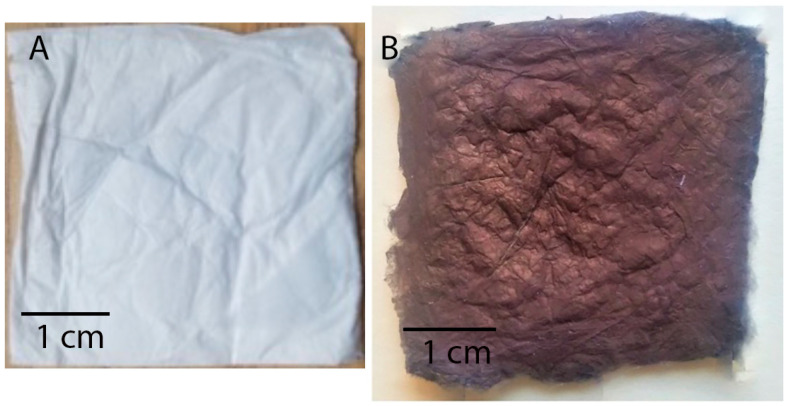
Optical images of example fiber mats: (**A**) a typical pristine PAN fiber mat and (**B**) electrolessly coated mat at conditions of near complete Cu coverage. The color of any given Cu coated mat roughly scales between these extremes with the amount of Cu coverage.

**Figure 3:**
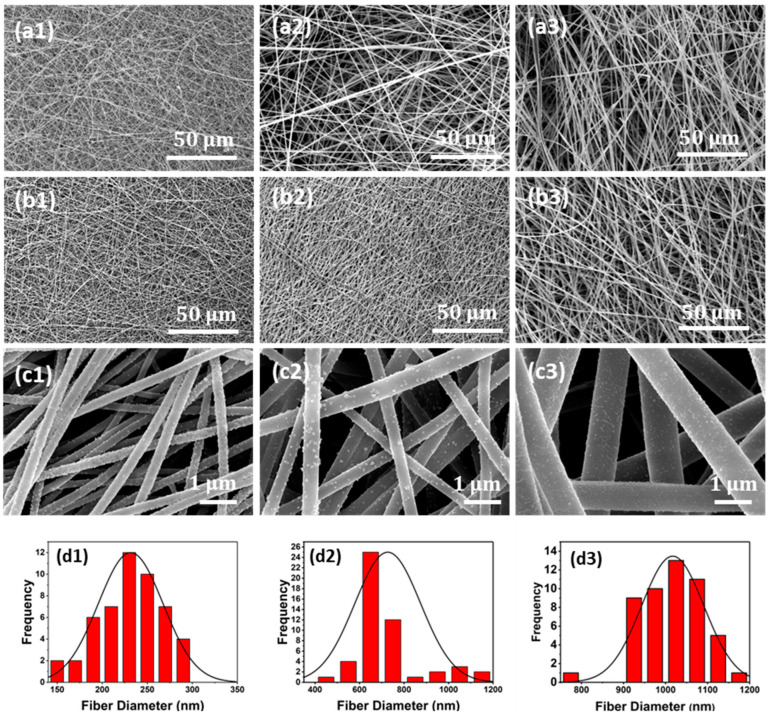
Representative microstructures of the pristine as grown and Cu NP metallized electrospun membranes. (**a1**) Pristine fiber mats with an average fiber diameter of 232 nm, (**b1**) 727 nm, and (**c1**) 1017 nm. (**a2**) Cu NP metallized fiber mats with average fiber diameter of 232 nm, (**b2**) 727 nm, and (**c2**) 1017 nm. (**a3**) High-magnification image for (**a2**); (**b3**) high-magnification image for (**b2**); and (**c3**) high-magnification image for (**c2**), all showing uniform coverage of isolated Cu NP on the fibers. (**d1**–**d3**) Histogram of fiber diameters.

**Figure 4:**
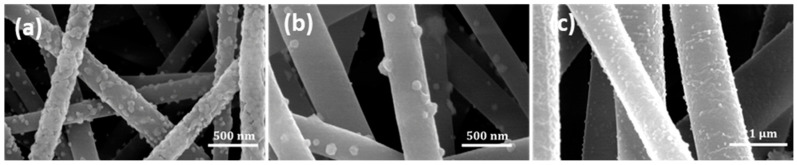
Higher magnification of samples showing well-dispersed, irregularly shaped Cu-NPs: (**a**) 232 nm average fiber diameter (AFD) mats; (**b**) 727 nm AFD mats; and (**c**) 1017 nm AFD mats.

The fiber assembly in electrospun mats is typically random, with the distribution dictated by the complex coupling of the applied electrostatic field and solution rheology during jet formation and travel during processing [35]. Overview optical images of a pristine mat and a mat coated with Cu NP at almost complete Cu coverage are shown in Figure 2. The morphology of the pristine and Cu NP–PAN fiber mats are shown in Figure 3. For PAN concentrations of 9 wt.%, 11 wt.%, and 13 wt.% in solution (while keeping invariant the relative composition of acetone in solution) the corresponding fiber sizes were 232 ± 36 nm, 727 ± 148 nm and 1017 ± 80 nm, which are shown in Figure 3(a1), (b1) and (c1), respectively. For PAN/DMF solutions (and most polymer systems), an increase in the concentration of PAN in solution causes an increase in the solution viscosity, which in turn leads to an increase in the resulting fiber diameters [36]. After the Cu NP metallization procedures, the open-cell architecture was preserved for all mats across the distinct fiber diameters, as evidenced by the SEM images in Figure 3(a2–c2). Furthermore, the Cu NPs had a discrete distribution on the fiber surface, as shown in Figure 3(a3–c3). However, a sparse distribution of submicron Cu particles was also observed. The apparent size of the NPs ranged from 20 to 140 nm. Morphologically, the nanoparticles were highly irregular and non-faceted, as can be observed in Figure 4, in contrast to the cubic crystal habit formed on aligned PAN fibers using a similar deposition protocol in our earlier work [17]. This structure difference may be due to deposition being a much faster process than surface diffusion in this case, due to the considerably higher density of nucleation or catalytic sites (a consequence of a greater number density of fibers).

This is consistent with observations of the influence of fiber density on nucleation sites and metallization kinetics during electroless copper deposition [19], wherein low number density fiber mats induced slower nucleation and deposition rates, and high number density fiber mats showed enhanced deposition rates.

**Figure 5:**
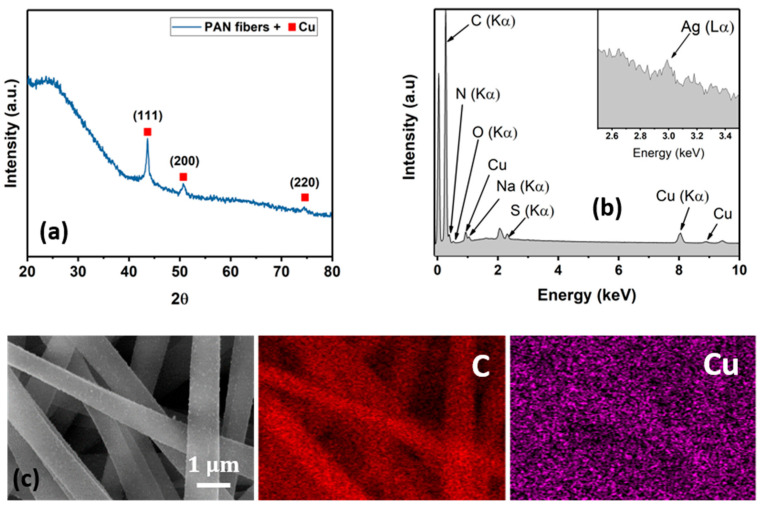
Materials characterization. (**a**) Representative diffraction pattern of the Cu NP–PAN Fiber mats. (**b**) EDX spectrum showing characteristic spectral lines of C, N, O, Cu, Na and S. The unmarked peaks are the Pt from the coating process. Na and S are possibly a result of the residual solvents from the electroless process. (**c**) Representative elemental maps of the Cu NP–PAN fiber mat.

Representative diffraction spectra, an EDX spectrum, and elemental maps of the Cu NP–PAN fiber mats are shown in Figure 5. For the diffraction pattern shown in Figure 5a, the broad peak between 20 and 35° is associated with the amorphous nature of PAN macromolecules. Characteristic copper peaks are observed at Bragg angles of 43.5°, 50.4°, and 74.6° for (111), (200) and (220) Cu planes, respectively (JCPDS Card No. 4-0836), revealing the crystalline nature of the deposited Cu species as well as their nanoparticulate dimensions given peak broadness. Characteristic silver peaks were not observed in the diffraction pattern, possibly due to a small scattering volume. However, the EDX spectrum shown in Figure 4b shows characteristic spectral lines of elemental silver. Elemental maps for carbon and copper are shown in Figure 5c, illustrating the sparse and disperse distribution of copper on the PAN fibers. The atomic weight percentages are summarized in Table 1.

### 3.2. FT–Infrared Spectroscopy

**Figure 6:**
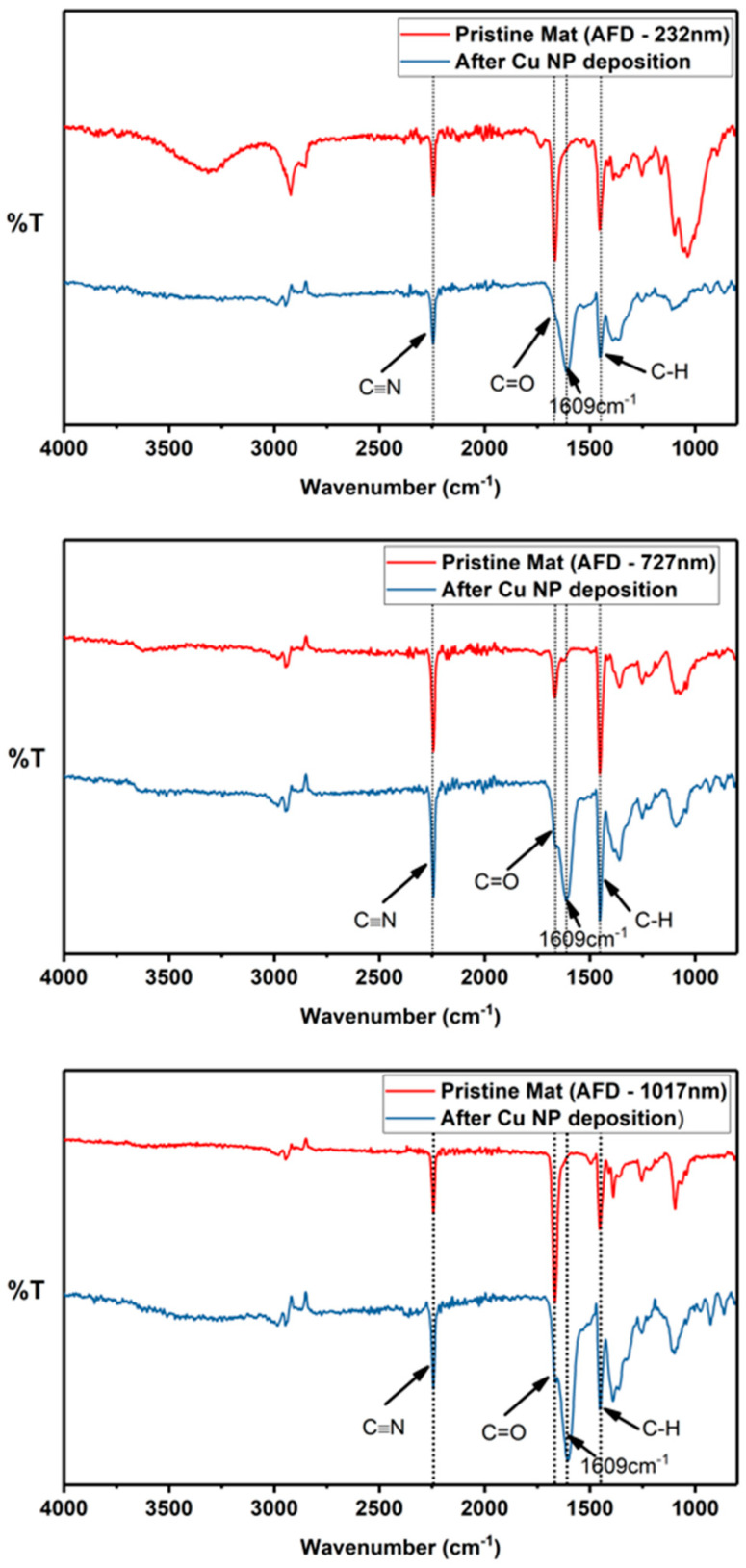
FTIR spectra of the electrospun PAN mats after electroless deposition with fiber sizes of (**top**) 232 nm, (**middle**) 727 nm, and (**lower**) 1017 nm. The intense peak at 1609 cm^−1^ observed for all metallized fibers is indicative of the cyclization of the nitrile functional groups, a feature linked to enhanced adhesion between metallic nanoparticles and PAN fibers.

The FTIR spectra of the PAN fiber mats before and after exposure to the chemical treatment protocols for electroless Cu NP deposition are shown in Figure 6. Broadly, pristine and Cu-deposited PAN fiber mats show signature PAN peaks at bands of 2240 cm^−1^ and 1451 cm^−1^, attributed to stretching of the cyano functional group, ν(C≡N), and bending in δ(C–H), respectively [37]. Furthermore, peaks at a wavenumber of 1665 cm^−1^ were also observed in the acquired spectra. These are indicative of stretching in a carbonyl group, ν(C=O) [35], likely a result of residual molecules of the carbonyl-containing solvents used for the electrospinning solution. Furthermore, upon Cu NP immobilization, the spectra of the fiber mats showed the evolution of a new peak at a wavenumber of 1609 cm^−1^, which has been assigned to the concurrent vibrational modes of the C=C, C=N and N−H bonds [38]. This suggests that a chemically induced cyclization of the nitrile functional groups in PAN molecules occurred during the metallization process, consistent with the spectral modifications observed after PAN fibers were subjected to similar metallization procedures using palladium-catalyzed copper deposition [39]. This cyclization process is proposed to be an important element of the working mechanism that enhances the adhesion between metallic nanoparticles (grown via electroless deposition) and PAN fiber substrates [17].

For all Cu-deposited PAN fiber mats, the undiminished intensity of the cyano band indicates that the cyclization is fiber surface restricted and the core (or bulk) of the fiber is largely unaffected.

### 3.3. Pore Size, Flux Measurement, and Filtration Performance

**Figure 7:**
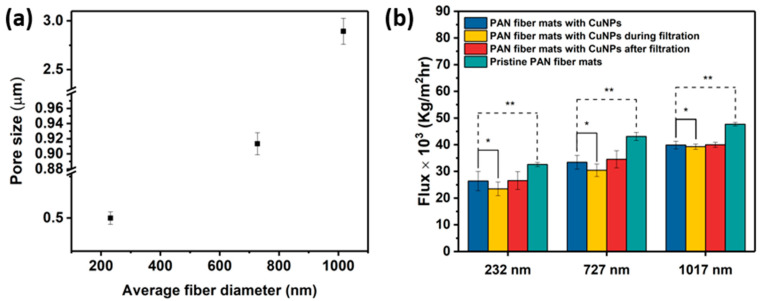
(**a**) Plots of pore size vs. average fiber diameter. (**b**) The flux of pure DI water and of DI water during and after the separation efficiency test with suspensions containing 100 ppm of 3 μm polystyrene beads. A single asterisk (*) indicates statistically insignificant differences between the flux of pure DI water and flux during separation test, while double asterisks (**) indicate statistically significant differences in the flux between pristine fiber mats and the corresponding Cu-metallized fiber mats.

The convoluted topology of electrospun fibers creates a highly complex pore structure in the mats. Figure 7a shows the effect of the fiber diameter on the pore size. It is observed that an increase in the constitutive fiber diameters increases the mean pore sizes of the electrospun mats. The mean pore sizes, summarized in Table 2, indicate that these mats are in the microfiltration range, i.e., 0.1–10 μm. Specifically, the corresponding pore sizes were greater by factors of ~2 and ~3 for mats with fiber diameters of 232 nm and 1017 nm, respectively, whereas a factor of 1.25 was observed for mats with fiber diameters of 727 nm. These relationships are within the predictions made for electrospun architectures, independent of polymer chemistry [40]. Nonwoven fibers differ from other designs because the pore geometry is not determined by definite shapes, in contrast to filtration membranes derived from other procedures. Instead, the pore size and distribution are a geometric consequence of the multiple fiber crossings and the attendant polygonal shapes formed. The polygon boundaries are the fiber segments between adjacent contact points, which can be deduced from Figure 3(c1–c3). Since the total number of fiber–fiber contact points in an electrospun fiber network increases with decreasing fiber diameter [41], the pore or “void” dimension is commensurately modulated. In accordance, the effective pore or “polygon” size decreases with increasing diameter, which explains the experimental observation.

The flux and separation efficiency measurements using 3 μm polystyrene particles are shown in Figure 7b. Polystyrene beads have been used to characterize the microfiltration efficacy of electrospun fibers [6]. Broadly, the pristine and Cu NP–PAN fiber mats allow a high flux. This is a result of the thin cross-sections of the membranes, which have been observed in electrospun polyimide fibers with a similar thickness and areal density as the mats in this study [42]. This dimensional effect is coupled with the fact that the mats are unconsolidated at fiber–fiber contact points. Water flux increased with an increase in mat fiber diameter, consistent with the accompanying increase in pore size. There was a 16–19% decrease in flux upon Cu NP metallization for all fiber sizes. This decrease may be attributed to a slight pore constriction by the deposited nanoparticles, or to a surface tension-mediated collapse of the fiber assembly during the aqueous electroless deposition process which could lead to relatively smaller pores.

The separation factors for a feed solution with 100 ppm of 3 μm model polystyrene were 99.7%, 99.40%, and 99.36% for Cu NP-metallized mats with fiber sizes of 232 nm, 727 nm, and 1017 nm, respectively. Table 2 shows that, in general, the fiber mats display high particle rejection/separation. This slightly reduces with increasing fiber size in a manner that is consistent with the measured pore sizes. There was a slight drop in flux during the particle separation test in the orders of 11%, 9%, and 2% for the metallized mats with average fiber diameters of 232 nm, 727 nm, and 1017 nm, respectively. This is attributed to the progressive particle entrapment and clogging of the pores during testing; a phenomenon that is worsened in microfiltration membranes with smaller fiber diameters [30]. Upon washing/rinsing of the membranes after the particle separation tests, the water flux recovered, signifying that potential membrane fouling can be reversible. Electrospun filtration membranes typically display good flux recoverability because of the interconnected pore structure, which is a distinguishing structural feature that contrasts with other established membrane designs [42,43]. It is important to note that, for each fiber size considered, the “clean” water flux and bead suspension flux through Cu NP-deposited mats are statistically similar (*p* > 0.05), implying that flux performance is not significantly diminished during separation/filtration. In essence, the Cu-NP loading does not lead to a significant impediment to water flow through the mats, signifying that the mat structures are largely preserved with very minimal blockages. In addition, the high flux and high separation efficiency indicate strong microfiltration performance with respect to the micron-sized particles/microbes.

**Figure 8:**
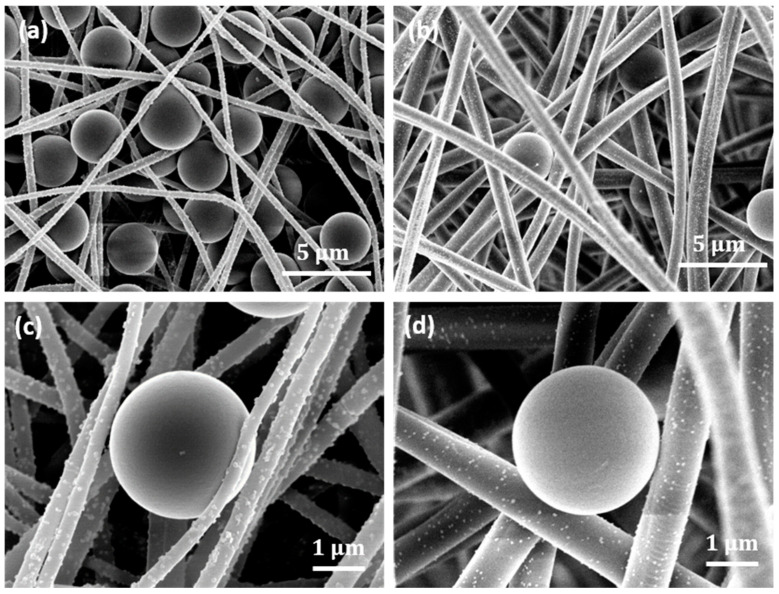
Representative images. (**a**) Trapped 3 μm polystyrene beads in the Cu-NP-deposited electrospun PAN mats with 282 nm fiber size. (**b**) Trapped beads in electrospun mats with 1017 nm fiber size. (**c**) A bead intercepted by 282 nm fiber size. The Cu NP-loaded fiber conforms to the beads, showing potential enhanced contact for antimicrobial effect. (**d**) Fibers of 1017 nm size, which conform less to the particle shape due to higher rigidity.

Figure 8 shows representative SEM images of the Cu NP–PAN fiber mats with trapped polystyrene beads after the filtration and washing steps, providing microstructural evidence for use as high-efficiency microfiltration membranes. The complex fiber architecture acts as a structural impediment to the passage of particles, intercepting and capturing the beads during flow. Figure 8a shows the entrapment of multiple beads by the nanometric fibers; the entrapped beads effectively reduce the pore size of the mats, further enhancing mat separation. In contrast, this aggregation effect is less pronounced in micron-sized fibers, as shown in Figure 8b. These may be responsible for the lower separation flux observed for the mats with nanometric fiber sizes (i.e., 232 nm and 727 nm), as shown in Figure 7b. In addition, in the context of toxicity to pathogenic microorganisms, this entrapment potentially ensures the close contact of those species with the antimicrobial copper nanoparticles, as shown in Figure 8c,d, enhancing the membrane disruption processes as well as cellular devastation. It can be observed that the copper nanoparticles are still adhered to the fiber surface after the filtration process. This shows that the particles can withstand the impact of fluid flow without dislodgement, indicating the durability of the structure.

## 4. Conclusions

Filtration membranes containing antimicrobial Cu nanoparticles were synthesized through the electroless deposition of Cu on electrospun PAN fiber mats of distinct fiber sizes. Electroless deposition offers a simple, versatile, and tunable methodology for a surface-confined deposition process. This contrasts with other processes where the nanoparticles are susceptible to being embedded in the polymer fiber, thus altering its structure [44]. Exposure to chemical baths for pretreatment and metallization did not substantially alter PAN chemistry, although nitrile group cyclization in PAN, presumably limited to the surface macromolecules, was observed. Filtration and flux performance was governed by the constitutive fiber diameters of the mats: the flux increased with an increase in fiber size due to an accompanying increase in the pore sizes, while the separation efficiency for polystyrene beads of average diameters of 3 μm slightly decreased with increasing mat fiber size. Overall, the filtration and flux performance metrics were excellent across the distinct fiber mats, showing high flux and high filtration efficiency. Advantageously, there was no significant reduction in Cu-NPs on the fiber surface after the separation test, indicating the maintenance of structural integrity upon possible deformation induced by hydraulic flow. Furthermore, it was shown that the membrane fouling by the particles was reversible by simple rinsing/washing after the separation test, with the flux prior to filtration restored. We have demonstrated a platform that, with further research on pathogenic efficacy, could enable Cu NP-deposited electrospun filtration membranes to be used as antimicrobial microfilters for the removal and destruction of pathogenic bacteria in water, either as stand-alone filters or integrated into other filtration infrastructures.

## Figures and Tables

**Table 1:** EDX-estimated weight percentages of main elements in Cu-NP-loaded fiber mat.

Element	Wt%
Carbon	96.1
Copper	3.0
Sodium	0.5
Sulfur	0.3
Silver	0.1

**Table 2:** Summary of DI water flux measurements as well as separation efficiency/factor.

Mat Fiber Diameter (nm)	Mean Pore Size (μm)	Pristine Mat DI Water Flux (kg/m^2^ h)	Cu-Metallized Mat DI Water Flux (kg/m^2^ h)	Efficiency Flux (kg/m^2^ h)	Separation Factor (SF) (%)	DI Water Flux after Efficiency Test (kg/m^2^ h)
232 ± 36	0.50 ± 0.04	32,600 ± 800	26,400 ± 3600	23,500 ± 2600	99.70 ± 0.13	26,600 ± 3400
727 ± 148	0.91 ± 0.02	43,100 ± 1500	33,500 ± 2600	30,400 ± 2400	99.40 ± 0.06	34,500 ± 3200
1017 ± 80	2.90 ± 0.13	47,700 ± 600	39,900 ± 1500	39,000 ± 1000	99.36 ± 0.3	40,000 ± 1000

## Data Availability

The original contributions presented in the study are included in the article/supplementary material, further inquiries or requests for raw data files can be directed to the corresponding author.

## Supplementary Materials

The following supporting information can be downloaded at https://www.mdpi.com/article/10.3390/membranes14090198/s1, Supplemental Figure S1. Electrospinning process flow.
