# Supplementary material for: Electroless Deposition for Robust and Uniform Copper Nanoparticles on Electrospun Polyacrylonitrile (PAN) Microfiltration Membranes
Source: Membranes (Basel). 2024 Sep 20;14(9):198. doi: 10.3390/membranes14090198 (PMC11434320; doi:10.3390/membranes14090198)
Supplement: Supplementary file 1 [file membranes-14-00198-s001.zip › membranes-3154441-supplementary.pdf]

Supplemental Figure S1
Electrospinning process flow for Aminu et al

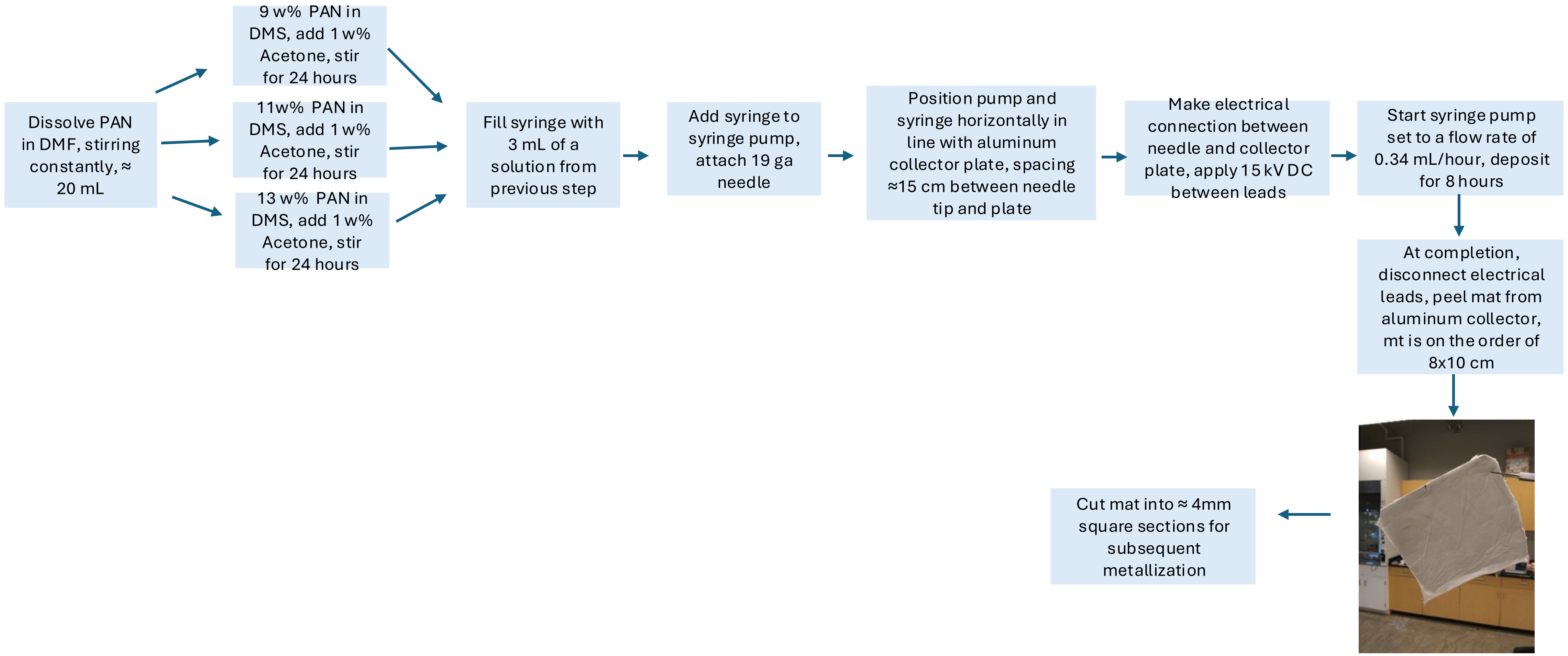

Figure S1. Process flow for electrospinning of the PAN fiber mat prior to metallization. Figure includes optical image of mat once separated from the aluminum collector plate.
